# Diffusion of Resveratrol in Silica Alcogels

**DOI:** 10.3390/molecules24213931

**Published:** 2019-10-31

**Authors:** Yuxiang Wang, Zeyu Kao, Ting Zhang, Yujun Zhang, Lili Qin, Zhihua Zhang, Bin Zhou, Guangming Wu, Jun Shen, Ai Du

**Affiliations:** 1 Shanghai Key Laboratory of Special Artificial Microstructure Materials and Technology, School of Physics Science and Engineering, Tongji University, Shanghai 200092, China; 1653553@tongji.edu.cn (Y.W.); 1653580@tongji.edu.cn (Z.K.); 1730965@tongji.edu.cn (T.Z.); zzhtj@tongji.edu.cn (Z.Z.); zhoubin863@tongji.edu.cn (B.Z.); wugm@tongji.edu.cn (G.W.); shenjun67@tongji.edu.cn (J.S.); 2 Sports and Health Research Center, Department of Physical education, Tongji University, Shanghai 200092, China; 1750466@tongji.edu.cn

**Keywords:** RLSA, one-dimension diffusion, alcogel, adsorption/desorption, Silica Aerogel

## Abstract

The trans-resveratrol (RSV)-loaded silica aerogel (RLSA) was prepared by the sol-gel method, adding the drug during the aging process, solvent replacement and freeze drying. A series of characterizations showed that RSV stays in the silica aerogel in two ways. First, RSV precipitates due to minimal solubility in water during the solvent replacement process. After freeze drying, the solvent evaporates and the RSV recrystallizes. It can be seen from scanning electron microscope (SEM) and transmission electron microscope (TEM) images that the recrystallized RSV with micron-sized long rod-shaped is integrated with the dense silica network skeleton. Second, from small-angle X-ray scattering (SAXS) results, a portion of the RSV molecules is not crystallized and the size is extremely small. This can be attached to the primary and secondary particles of silica to enhance its network structure and inhibit shrinkage, which is why the volume and pore size of RLSA is larger. In addition, the diffusion of RSV in silica alcogel was studied by a one-dimensional model. The apparent diffusion coefficients of inward diffusion, outward diffusion and internal diffusion were calculated by fitting the time- and position-dependent concentration data. It was found that the outward diffusion coefficient (5.25 × 10^−10^ m^2^/s) is larger than the inward (2.93 × 10^−10^ m^2^/s), which is probably due to the interface effect. The diffusion coefficients obtained for different concentrations in the same process (inward diffusion) are found to be different. This suggests that the apparent diffusion coefficient obtained is affected by molecular adsorption.

Academic editor: Alejandro Sosnik

## 1. Introduction

Drug-delivery systems are difficult to design as there exist various mechanisms that are involved in the release processes of drug [1,2]. Various factors need to be considered for drug delivery, such as the degree of absorption or the diffusion limitations of the drug in the medium [3]. In recent years, the use of porous silica materials in drug delivery has attracted a lot of interest due to its potential for the development of systems that are site-specific and that offer time-dependent controlled delivery [3,4,5]. Given in particular their non-toxic properties, silica aerogels are a special kind of porous silica materials consisting of highly mesoporous nanostructures. The porous structure make the aerogels have many special properties [6], such as low density (1000 kg/m^3^ to about 1 kg/m^3^) [7], high porosity (~99%) [8] and inner surface area (1000 m^2^/g), which is necessary for efficient drug loading. In addition, silica aerogels can be grafted with various groups by surface modification, which could deliver the drug to targeted regions [9,10,11].

Trans-resveratrol (RSV), whose full name is non-flavonoid phytoalexin trans-resveratrol, has many biological functions by virtue of its antibacterial, anti-inflammatory, anticancer, anti-thrombosis, anti-hyperlipidemia and anti-lipid peroxidation characteristics. These functions determined resveratrol has many supplementations in treatment of cancer, diabetes, skin disorders and so on [12,13]. In this paper, silica aerogel and RSV was chosen as a carrier and a model drug, respectively. Drug-delivery vehicles based on aerogels can be prepared by different methods, such as the addition of the drug during the conventional sol–gel process or during the post treatment of the synthesized aerogels [1,14]. We added the drug during the aging process in this work. The alcogel was brought into contactwith an aging solution which contains the RSV, and then the drug diffused into the alcogel pores from the aging solution. The diffusion rate depends on many factors, such as the size of the molecules and pores, and the initial concentration of the aging solution. It must be emphasized that the role of ethanol was simply to dissolve the drug and allow it to diffuse into the alcogel.

Most research focuses only on the total drug loading content, but ignores the drug distribution and load mode in the carrier. Moreover, drug-loading behaviors are commonly studied using adsorption isotherms and adsorption kinetic equations [15]. However, diffusion also play an important role in the drug-loading process, and we should consider both the effects of diffusion and adsorption/desorption. So in this work, based on previous study, we have established a fundamental model to study the distribution of a drug in the alcogel and the interaction of diffusion and adsorption/desorption [15].

In this paper, RSV-loaded silica aerogel (RLSA) was prepared by freeze-drying. The reason we used freeze drying instead of supercritical drying is that the RSV will lose ethanol during the slow replacement process in supercritical drying. A series of characterizations were carried to compare the differences of silica aerogel and RLSA, and better understand the loading mode of the drug in silica aerogel. Furthermore, by establishing different boundary conditions and using Fick’s second law to fit the apparent diffusion coefficient (the apparent diffusion coefficients are all marked as “diffusion coefficient” in the manuscript hereafter for simplicity), three kinds of diffusion processes were studied. This facilitates the precise design of the drug-loading and -release system.

## 2. Results and Discussion

### 2.1. The Characterization of Silica Aerogel and Trans-Resveratrol (RSV)-Loaded Silica Aerogel (RLSA)

**Figure 1:**
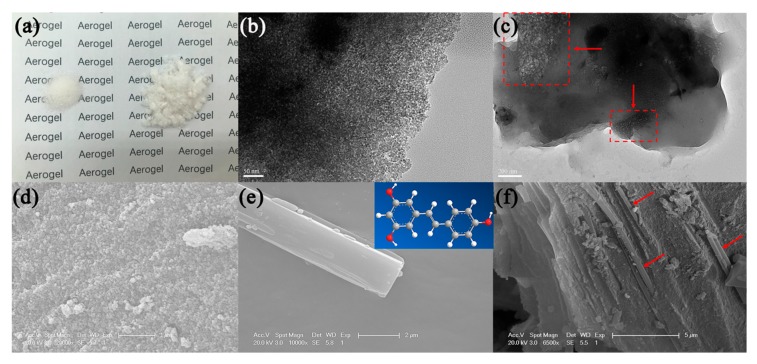
(**a**) The appearance of silica aerogel (left) and trans-resveratrol (RSV)-loaded silica aerogel (RLSA) (right) after freeze drying; (**b**,**c**) Transmission electron microscope (TEM) images of silica aerogel and RLSA; (**d**–**f**) Scanning electron microscope (SEM) images of silica aerogel, RSV and RLSA.

Figure 1a shows the appearance of silica aerogel and RLSA after freeze drying. The mass of both is 200 mg. We can see that the volume of the RLSA is larger than silica aerogel, and the color becomes more opaque. A transmission electron microscope (TEM) image of silica aerogel shows that it is a dense structure formed by the accumulation of spherical particles, which is consistent with a scanning electron microscope (SEM) image. Compared with the silica aerogel after supercritical drying, its skeleton structure is denser and the density is larger. However, the pore size is smaller and most of it consists of micropores [16]. The structure of RSV has a long rod shape with the size on the order of micrometers shown in Figure 1e. It can be seen from Figure 1c,f that the recrystallized RSV with a micron-sized long rod shape is integrated with the dense silica network skeleton. This is may be due to the RSV dissolving into small molecules in an ethanol solution, which can diffuse into the pores of silica alcogel. However, RSV precipitates due to minimal solubility in water during solvent replacement. After freeze drying, the solvent evaporates and the RSV recrystallizes.

**Figure 2:**
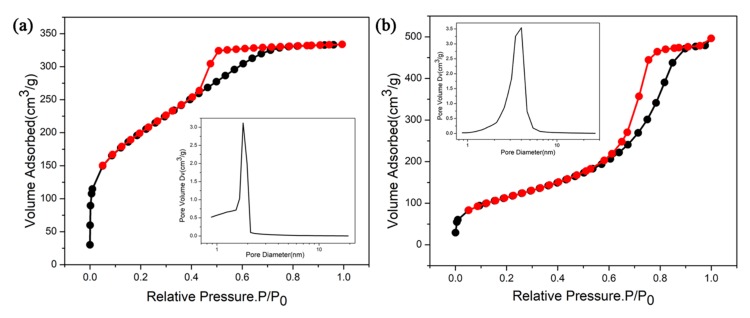
(**a**,**b**) The N_2_ adsorption-desorption isotherms and the pore size distributions of silica aerogel and RLSA, respectively.

Figure 2a,b shows the N_2_ adsorption–desorption isotherms and the pore size distributions of silica aerogel and RLSA, respectively. Figure 2a presents an I-type curve and most microporous materials belong to this category. The pore structure of the H4-type is a wedge-shaped hole piled up in the skeleton, but more pores are concentrated in the microporous area [17]. We can see that the pore size of silica aerogel is concentrated in micropores with an average value of 1.51 nm. The specific surface area is 702 m^2^/g. This is consistent with the dense structure exhibited by the SEM and TEM images. Figure 2b presents an IV-type curve illustrating that RLSA contains mesoporous elements. H1-type hysteresis loop indicates that there are a large number of spherical pores stacked in succession [18]. The average pore size of RLSA is 3.24 nm and most pores are distributed in the region of the micropores or small mesopores. Its specific surface area is 411 m^2^/g. Therefore, it is presumed that the small drug molecules are not left in the silica aerogel in a pore-filling manner. If so, the pore size of the RLSA should be smaller.

**Figure 3:**
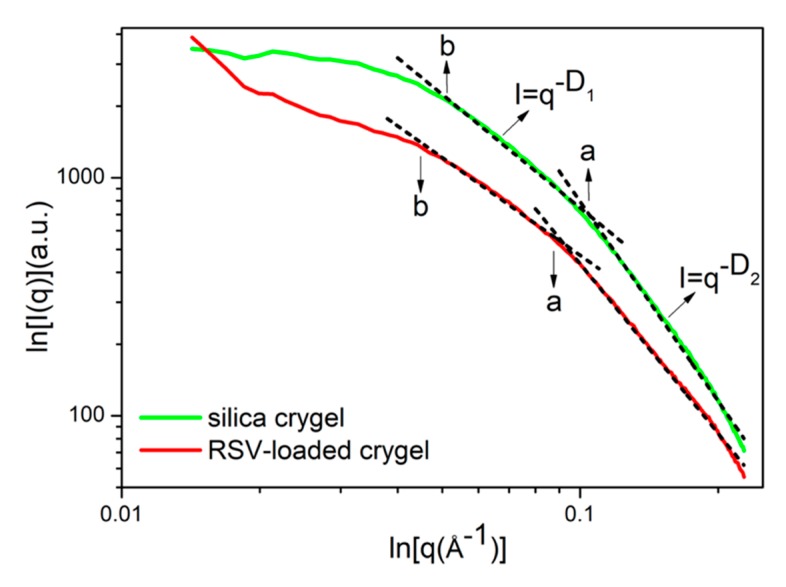
The small-angle X-ray scattering (SAXS) image of silica aerogel and RLSA, respectively.

In order to understand the drug loading mode of silica aerogel after freeze drying, small-angle X-ray scattering (SAXS) of the two samples were characterized. As shown, both the silica aerogel and the RLSA have no surface fractals. The mass fractal dimensions D_1_ of two samples are between 1–2 in Table 1 and Figure 3, indicating that the internal character is a chain structure formed by primary particles and clusters. The fractal dimension D_2_ of the silica aerogel is 2.79, which indicates that the primary structure is relatively dense. The larger the value, the denser the system, which may be related to the volume shrinkage during freeze drying. In the RLSA system, the mass fractal dimension D_2_ is 2.37. The smaller value shows that the mass contained in the unit volume of the fractal structure is reduced, and the system is looser. However, the size of the primary particles “a” is larger than that of silica aerogel. This may be due to the adhesion of small drug molecules to the silica particles, enhancing their network backbone structure. This will make the primary and secondary particles larger in the RLSA system. Also, the reinforcement of the skeleton inhibits shrinkage during freeze drying, making the volume and the pore size of the RLSA larger. The smaller mass fractal dimension of the RLSA than the silica aerogel also indicates that the system is not uniform.

**Figure 4:**
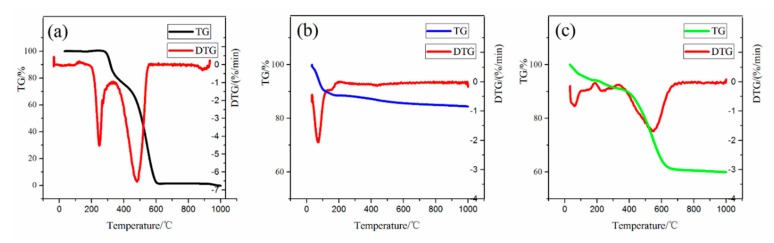
The TG-DTG (Thermogravimetric-Derivative Thermogravimetric) curves of (**a**) RSV, (**b**) silica aerogel and (**c**) RLSA.

A thermogravimeter (TG) is used to measure the drug loading content in silica aerogel. Figure 4a shows that the RSV starts to decompose from 280 °C and reaches the extreme value of the weight drop rate at 315 °C and 550 °C. It completely decomposes until 610 °C [19]. From Figure 4b, the aerogel has a weight loss of 2.6% in the RSV decomposition temperature range. This is mainly attributed to the liberation of surface-adsorbed water [20,21]. Figure 4c shows that the RLSA has a weight loss of 28.5% in the same range. This value represents the amount of surface-adsorbed water and the RSV. Therefore, the RSV loading content was 26.7%. The volume of silica alcogel is 25 mL and it becomes powdery after drying with the weight of 1.6 g. The soaking concentration is 13.3 g/L so the expected drug loading content is 0.025 L × 13.3 g/L = 0.33 g. However, the actual drug loading measured by the TG is 1.6 g × 26.7% = 0.43 g. This may be because the RSV-EtOH concentration used in the fitted diffusion coefficient experiment (as described below) is much lower than RLSA (40 g/L). After the concentration of the solution was increased, the effect of adsorption was enhanced, which caused more drug are adsorbed on the skeleton network. Therefore, the adsorption and diffusion work together to make the actual drug-loading content greater than in theory.

### 2.2. Inward Diffusion Coefficient

The cuvette alcogel of the experimental group was immersed into a certain concentration of RSV-EtOH solution. The volume of the RSV-EtOH solution is larger compared to the alcogel, so we approximate that the concentration of this solution is constant. Since the concentration of RSV in the alcogel varies with time and position, Fick’s second law was used to fit it. The diffusion differential equation of Fick’s second law is:

(1) $$\frac{\partial c\left(x,t\right)}{\partial t}=D\frac{\partial^{2}c\left(x,t\right)}{\partial x^{2}}$$

The variable *c*, a function of position *x* and time *t*, is the concentration of RSV in alcogel. The top of cuvette was defined as position origin. Constant L and *n*_0_ are the length of alcogel in cuvette and the concentration of RSV solution, respectively. The initial condition is:

(2) c(x,0)=0, 0<x<L

The boundary conditions are:

(3) $$c\left(0,t\right)=n_{0},\;\frac{\partial c\left(L,t\right)}{\partial x}=0$$

We can derive series solution after detaching variables:

(4) $$c\left(x,t\right)=n_{0}\left[1-\overset{\infty }{\underset{n=0}{\sum }}\frac{2}{\left(n+1/2\right)\pi }e^{-{\left(\frac{n+1/2}{L}\pi \right)}^{2}Dt}sin\left(\frac{n+1/2}{L}\right)\pi x\right],\;0<x<L$$

**Figure 5:**
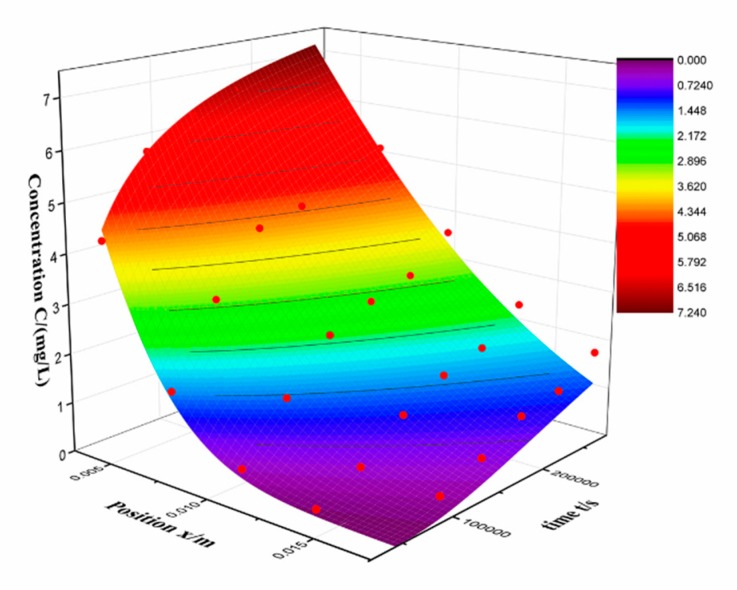
Inward diffusion original experimental data-fitting surface.

The measurement data is fitted using a loop statement fitting program. The value of *n* ranges from 0 to 500. The results are shown in Table 2 and Figure 5.

The value of D and R^2^ is 2.928 × 10^−10^ m^2^/s and 0.98, respectively. The result fits well with Fick’s law, indicates that the diffusion is main process of RSV entering to the silica alcogel. The value of diffusion coefficient that Fe^2+^ enter the silica gels is 3.52 × 10^−7^ m^2^/s in our previous work [17], which is far larger than RSV. This is consistent with our expectations, ions are easier to diffuse than organic molecules because of smaller volume. It can be seen in the fitted graph that the measured data (red dot) is above the fitted surface at low concentration and below the fitted surface at high concentration. Actually, it is found that the residual is far larger in low concentration. Table 3 shows the concentrations and residuals at third day and the percentage represents the ratio of the two. The residual is maximal at *x* = 17.5 mm. In order to minimize the error, the experiment was repeated several times. So, the residual indicates that the inward loading process is not only a diffusion process but an adsorption process. Under the same measurement conditions, the larger the residual, the stronger the diffusion and adsorption coupling. Thus, the inward diffusion coefficient can describe the diffusion process of adsorption coupling. And the residual can represent the coupling strength.

In view of the above results, different ranges of the concentrations in the same experiment were selected to fit the diffusion process. All concentration data of position A, B and D, E are considered as high C and low C, respectively. This result shows that the diffusion coefficient we measured is an apparent value that affected by adsorption, rather than the true diffusion coefficient. The concentration of RSV that used to fit the diffusion coefficient consists of two parts. One is the RSV adsorbed on alcogel skeleton, the other is in the solvent inside pores. These two parts correspond to the adsorption and diffusion processes, respectively. The diffusion coefficient is the result of the superposition of the two, reflecting the coupling relationship between adsorption and diffusion [22]. From this we speculate that at lower concentrations, it is caused by both adsorption and diffusion. Therefore, under the interference of adsorption, the correlation coefficient R^2^ is also low as in Table 4. However, at high concentrations, the adsorption reaches saturation and only diffusion occurs, which making the diffusion rate lower, but the correlation coefficient R^2^ is higher. However, in general, the adsorption is very weak compared to diffusion. The correlation coefficient R^2^ in Table 1 is as high as 0.98, indicating that the process is dominated by diffusion [23,24].

### 2.3. Outword Diffusion Coefficient

An outward diffusion model was built which is similar to inward diffusion. The inward diffusion model reaches a diffusion equilibrium after a long period of time. Diffusion equilibrium, that is, the concentration of RSV is equal throughout the alcogel. The difference between inward diffusion and outward diffusion lies in the direction. Fick’s second law is as follows:

(5) $$\frac{\partial c\left(x,t\right)}{\partial t}=D\frac{\partial^{2}c\left(x,t\right)}{\partial x^{2}}$$

The initial condition is:

(6) $$c\left(x,0\right)=C_{0},\;0<x<L$$

The boundary conditions are:

(7) $$c\left(0,t\right)=0,\;\frac{\partial c\left(L,t\right)}{\partial x}=0$$

We can derive series solution after detaching variables:

(8) $$c\left(x,t\right)=C_{0}\overset{\infty }{\underset{n=0}{\sum }}\frac{2}{\left(n+1/2\right)\pi }e^{-{\left(\frac{n+1/2}{L}\pi \right)}^{2}Dt}sin\left(\frac{n+1/2}{L}\pi x\right),\;0<x<L$$

**Figure 6:**
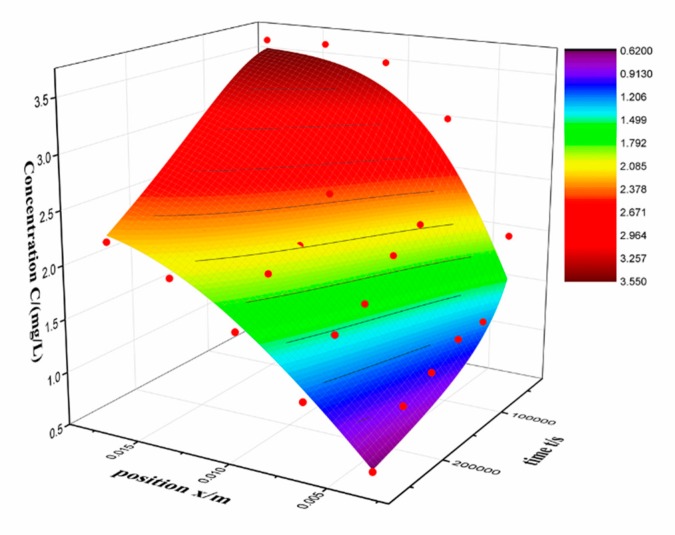
Original experimental data and fitted surface of outward diffusion.

This is similar to formula (4). What we need to focus on is that the initial conditions and boundary conditions are different from those in 2.2. The fitting results are shown in Table 5 and Figure 6.

It can be seen that the outward diffusion coefficient was larger than the inward coefficient. We speculate that there may be an interfacial effect that hinders the inward diffusion of the drug. Since there is a hindering influence, it is suspected that the concentration of RSV near the alcogel interface may be lower than the solution. Therefore, the boundary condition *n*_0_ is taken as a parameter, and the inward diffusion experimental data was fitted again. The fitting results are shown in Table 6.

The value of *n*_0_ is approximately a constant in 2.2, but the fitted *n*_0_ is lower than the constant. This result shows a decrease in the RSV concentration at the alcogel interface of the inward diffusion process. The same idea is applied to the outward diffusion process, but this process is to take the initial condition C_0_ as a parameter. The fitted C_0_ is basically consistent with the controlled initial condition. Therefore, the interface may only hinder inward diffusion, and outward diffusion is almost unaffected. In other words, the difference of the diffusion coefficient may be induced by the interface effect. The diffusion from a non-dense medium (alcohol) to a dense medium (alcogel) is probably relatively difficult, leading to a lower diffusion coefficient. In practical applications, the apparent outward diffusion D can reflect the effect of sustained release. In the case of the same particle size, the smaller the D value, the longer the release time. According to Fick’s law, we can also get drug release curve by calculation.

### 2.4. Internal Diffusion Coefficient

It takes a long time for RSV to be uniformly distributed in the alcogel. If the diffusion coefficient is known, a drug-loading method can be designed. First, alcogel was soaking in a high-concentration drug solution. After *t*_1_ time, the average concentration in the alcogel can be calculated using Equation (9).

(9) $$\int_{0}^{L}c_{1}\left(x,t_{1}\right)dx=Lc_{0}$$

This step is inward diffusion. The *c*_1_*(x, t)* and *c*_0_ is the concentration of inward diffusion solution and designed concentration, respectively. After *t*_1_, the total mass of drug that has reached designed mass L*c_0_, the inward diffusion process should be stopped. The total mass can also be calculated by integration of concentration toward position *x*. Second, the alcogel is separated and start the internal diffusion process until the drug is evenly distributed. In theory, uniform diffusion takes an infinite amount of time. Yet, the difference between maximum and minimum concentration at different positions could describe the non-uniformity. If this difference could be ignored, the distribution could be considered to be uniform. So, internal diffusion time *t*_2_ was derived by Equation (10):

(10) $$Max\left[c_{2}\left(x,t_{2}\right)\right]-Min\left[c_{2}\left(x,t_{2}\right)\right]<eps$$

The *c*_2_*(x, t)* is the concentration after internal diffusion, whose initial condition is *c*_1_(*x, t*_1_). *eps* is the fitting accuracy requirements for uniformity. 

Internal diffusion occurs inside the alcogel which has a concentration gradient. The alcogel doesn’t contact with external. Correspondingly, the Neumann boundary conditions are 0 at both ends. The diffusion differential equation is:

(11) $$\frac{\partial c\left(x,t\right)}{\partial t}=D\frac{\partial^{2}c\left(x,t\right)}{\partial x^{2}}$$

The initial condition is:

(12) c(x,0)=F(x), 0<x<L

The boundary conditions are:

(13) $$\frac{\partial c\left(0,t\right)}{\partial x}=0,\;\frac{\partial c\left(L,t\right)}{\partial x}=0$$

**Figure 7:**
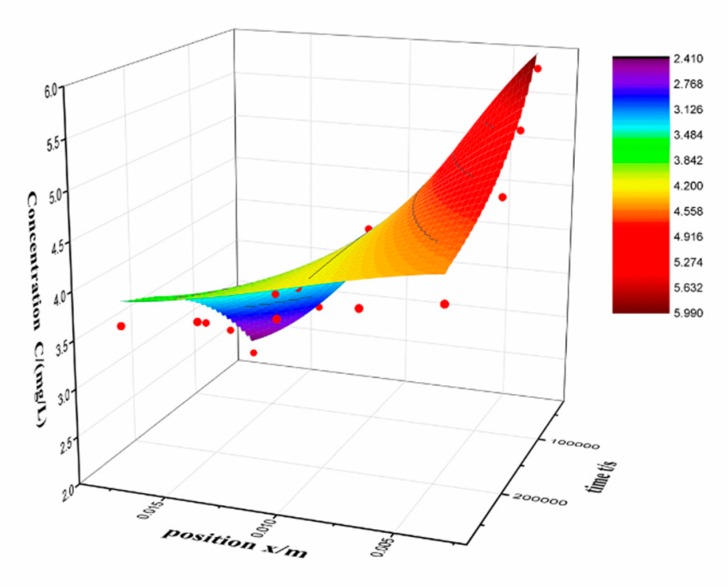
Original experimental data and fitted surface of internal diffusion.

*F(x)* is the initial concentration distribution in alcogel. Then we done the interpolation and solved the differential equation by finite element method with computer [25,26]. A numerical solution was used to fitted internal diffusion coefficient (Table 7 and Figure 7).

The internal diffusion process is the transition of drug from a higher concentration position to a lower. Desorption occurs at higher concentration and adsorption occurs at lower concentration. Therefore, the value of internal diffusion D is between outward diffusion and inward diffusion. At the same time, the drug does not pass through the interface during this process and there is no need to consider the interface effect.

In practical applications, the following constants are known: the size of the alcohol gel, the drug concentration C of the soaking solution, the desired *eps* and the inward diffusion coefficient D (D can be measured toward a special combination of drug and alcogels). The soaking time *t*_1_ and the internal diffusion time *t*_2_ (parameters *t*_1_ and *t*_2_ are computer calculation results) can be calculated. Thereby, the concentration of the drug in the alcogel is relatively quantitatively controlled.

## 3. Experiments and Characterizations

### 3.1. Materials

Tetraethylorthosilicate (≥28.4%, TEOS), ethanol (≥99.7%, EtOH), hydrochloric acid (36.0–36.8 wt%, HCl), ammonia hydroxide (25~28 wt%, NH_4_OH) were purchased from Sinopharm Chemical Reagent Co., Ltd., Shanghai, China. Resveratrol (≥99%, RSV) was purchased from Aladdin Reagent (Shanghai, China) Co., Ltd.

### 3.2. Preparation of Silica Gels

Silica alcogels were prepared by the traditional sol-gel method [7]. The TEOS, EtOH, hydrochloric acid and ammonia hydroxide were used as precursor, solvent and catalyst, respectively. The volume ratio of TEOS, EtOH, hydrochloric acid and deionized water was 1:0.5:1.5 × 10^−4^:0.083, named solution A. And the EtOH, deionized water, ammonia hydroxide was mixed at a volume ratio of 2.5:0.1:0.058, named solution B. After 30 min of stirring separately, solution B was poured into solution A. The sol was poured into the mold and place at room temperature after stirring for about 15 min.

### 3.3. One-Dimensional Diffusion Model

**Figure 8:**
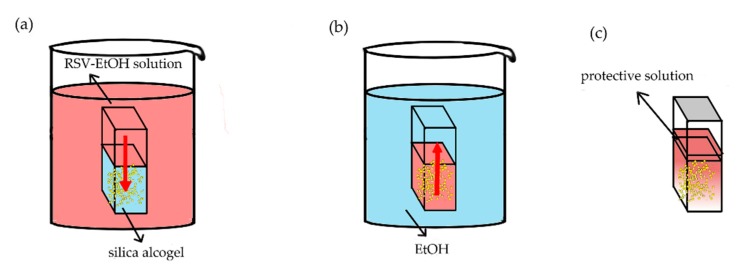
The schematic diagram of: (**a**) inward diffusion, (**b**) outward diffusion and (**c**) internal diffusion.

Silica alcogels were prepared in cuvettes and divided into two groups. One is negative control group with the size of 1 × 1 × 4 cm^3^; the other is experimental group with the size of 1 × 2 × 4 cm^3^. The optical path is 1 cm. In an experimental group cuvette, the alcogel is divided into six positions (in turn A, B, C, D and E, respectively, and the position A in contact with the RSV-EtOH solution) on average to measure the concentration. A schematic diagram of three diffusion models is shown in Figure 8. In particular, only the bottom surface of the cuvettes was in contact with the RSV-EtOH solution and the other surfaces were closed. Therefore, this can be considered a one-dimension diffusion model.

#### 3.3.1. Inward Diffusion

**Figure 9:**
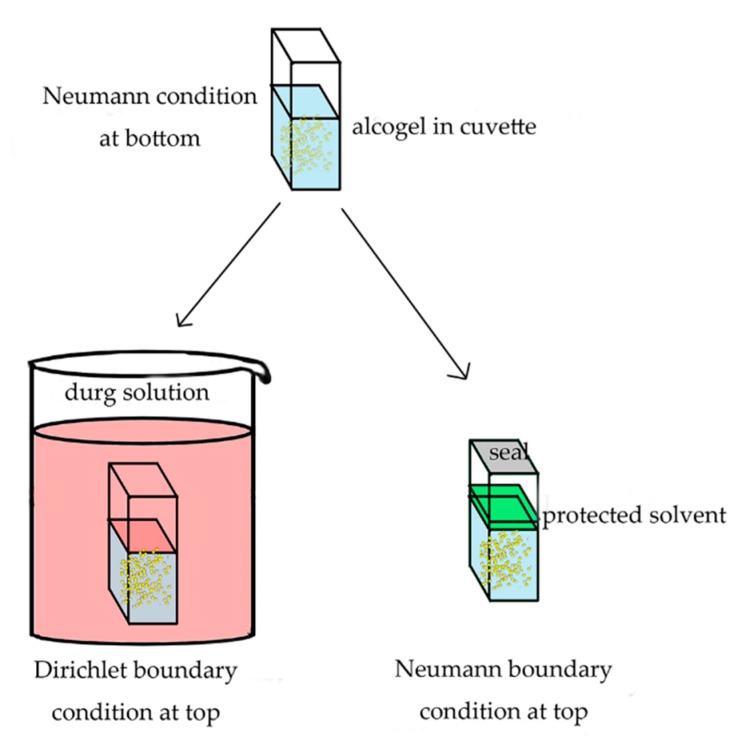
One-dimension diffusion model: prepare alcogels in cuvettes and kinds of boundary conditions.

The negative control cuvette was soaked in ethanol. The bottom of the experimental cuvette met the Neumann boundary condition due to the isolation by cuvette (Figure 9). The top of the experimental cuvette met the Dirichlet boundary condition when soaked in the RSV-EtOH solution. If the experimental cuvette was protected by a thin layer and sealed, the top was approximately the Neumann boundary condition. The experimental cuvette was soaked in the RSV-EtOH solution with the volume of 500 mL, far more than the volume of alcogels in cuvettes. Thus, the concentration of RSV in solution could be considered as a constant. Because the RSV is unsteady under light and high temperature, the solution was shaded and put in a constant temperature room (23 °C). This process is named RSV inward diffusion.

#### 3.3.2. Outward Diffusion

The inward diffusion process takes a long time to reach diffusion equilibrium, that is, when the RSV concentration of each positions of the alcogel is equal. RSV-EtOH solution is replaced with pure ethanol solution, and the RSV inside the alcogel begins the outward diffusion process. It is worth noting that pure ethanol should be replaced frequently.

#### 3.3.3. Internal Diffusion

The third experiment involved two processes: inward diffusion and internal diffusion (in particular, the premise of internal diffusion here is to isolate the outside world). After three days of inward diffusion, the cuvette was taken out of the RSV-EtOH solution. Leave a little RSV-EtOH solution at the alcogel interface to prevent the gel cracking, seal the cuvette and place under the same conditions. Meanwhile, the RSV inside the alcogel begins to internal diffusion. It should be noted that after three days of inward diffusion (that is, at the beginning of internal diffusion), the concentration of RSV inside the alcogel is gradient distributed.

### 3.4. Measurement of Concentration of RSV

The RSV in ethanol has a strong absorption peak at 306 nm. Therefore, the concentration of RSV can be calculated by the absorptivity according to the Lambert–Beer law. The negative control cuvette was used for comparison during the measurement. Before the experiments, the zero-point calibration was done when the concentration of both negative control cuvette and experimental cuvette were zero.

### 3.5. Freeze-Drying Process

The cylindrical alcohol gel was soaked in a 40 g/L RSV-EtOH solution, and the alcoholate was masked with tin foil. After four days, replace it four times with deionized water every 12 h. The hydrogel was then frozen at 20 °C for 24 h. Finally, the hydrogel was freeze-drying for 24 h at a temperature of 60 °C and a pressure of less than 100 Pa.

### 3.6. Characterizations

The microstructure and morphology of the silica aerogel, RSV and RLSA was characterized by scanning electron microscope (SEM, Philips-XL30FEG, Thermo Fisher Scientific Inc., Hillsboro, OR, USA) and transmission electron microscopy (TEM, */JEM-2100F, JEOL, Tokyo, Japan). The Brunner-Emmet-Teller (BET, AUTOSORB-1-MP, Quantachrome, Boynton Beach, FL, USA) was used to measure the pore-size distribution and specific surface area of the silica aerogel and RLSA. The small angle X-ray scattering (SAXS) test uses Cu target X-rays and the device is NanoSTR (Bruker-AXS, Germany). The actual drug loading is measured by a synchronous thermal analyzer (TG, STA449C, Netzsch, Selb, Germany). The absorption spectrum was measured by an ultraviolet-visible-infrared (UV-Vis-IR) spectrophotometer (JASCO V-570, JASCO, Kyoto, Japan).

## 4. Conclusions

In this work, the RLSA was prepared by the sol-gel method, adding drug during aging process, solvent replacement and freeze drying. A series of characterizations showed that RSV stays in the silica aerogel in two ways. The first is that RSV precipitates and recrystallizes in the silica network backbone with a micron-sized long rod shape. In addition, a portion of the uncrystallized small molecules attach to the primary and secondary particles of the silica, enhancing its network and inhibiting shrinkage. In addition, the diffusion of RSV in silica alcogels was studied by one-dimension model. The three diffusion coefficient were calculated by fitting the time- and position-dependent concentration data. It was found that the outward diffusion coefficient (5.25 × 10^−10^ m^2^/s) is larger than the inward (2.93 × 10^−10^ m^2^/s), which is probably due to the interface effect. The diffusion coefficient of different concentrations in same process (inward diffusion) was also calculated, and it was found to be related to the adsorption effect. Finally, a model was established to control the concentration of drug loading precisely.

## Figures and Tables

**Table 1:** Fitting results of SAXS data.

Samples	D_1_	D_2_	a/*nm*	b/*nm*
Silica aerogel	1.58	2.79	0.96	2.02
RLSA	1.36	2.37	1.20	2.35

**Table 2:** Fitting result of inward diffusion coefficient.

D (*m^2^/s*)	R^2^	*n*_0_ (*mg/L*)	L (*mm*)
2.93 × 10^−10^	0.98	9.50	34

**Table 3:** Residual at third day of inward diffusion.

*x* /*mm*	3.5	7.0	10.5	14.0	17.5
C *mg/L*	7.04	5.23	3.71	2.52	1.91
residual	−0.17	0.05	0.11	0.07	0.27
percentage	−2.4%	1.0%	3.0%	2.8%	14.3%

**Table 4:** Fitting result of inward diffusion coefficient at different C range with same data.

	D (*m^2^/s*)	R^2^	C Range (*mg/L*)	Positions (*mm*)
low C	3.34 × 10^−10^	0.89	0.18–2.52	14.0 and 17.5 (D and E)
high C	2.64 × 10^−10^	0.98	1.54–7.04	3.5 and 7.0 (A and B)

**Table 5:** Fitting result of outward diffusion coefficient.

D (*m^2^/s*)	R^2^	C_0_ (*mg/L*)	L (*mm*)
4.25 × 10^−10^	0.94	3.56	20

**Table 6:** Fitting result of inward and outward diffusion when treating boundary and initial conditions as parameters.

**Inward D (*m^2^/s*)**	**R^2^**	**Fitted *n*_0_ (*mg/L*)**	***n*_0_ (*mg/L*)**
3.46 × 10^−10^	0.99	8.61	9.50
**Outward D (*m^2^/s*)**	**R^2^**	**Fitted C_0_ (*mg/L*)**	**C_0_ (*mg/L*)**
4.27 × 10^−10^	0.93	3.57	3.56

**Table 7:** Fitting results of inward diffusion D and internal diffusion D.

	D (*m^2^/s*)	R^2^	L (*mm*)
Inward diffusion	2.57 × 10^−10^	0.98	20
Internal diffusion	3.38 × 10^−10^	0.96	20
